# The Effect of Sprouting in Lentil (*Lens culinaris*) Nutritional and Microbiological Profile

**DOI:** 10.3390/foods9040400

**Published:** 2020-04-01

**Authors:** Carla S.Santos, Beatriz Silva, Luísa M.P.Valente, Sabine Gruber, Marta W.Vasconcelos

**Affiliations:** 1 Universidade Católica Portuguesa, CBQF—Centro de Biotecnologia e Química Fina—Laboratório Associado, Escola Superior de Biotecnologia, Rua Diogo Botelho 1327, 4169-005 Porto, Portugal; 19.beatriz.98@gmail.com (B.S.);; 2 CIIMAR – Centro Interdisciplinar de Investigação Marinha e Ambiental, Universidade do Porto, Avenida General Norton de Matos, 4450-208 Matosinhos, Portugal; lvalente@icbas.up.pt; 3 ICBAS, Instituto de Ciências Biomédicas de Abel Salazar, Universidade do Porto, Rua de Jorge Viterbo Ferreira, 228, 4050-313 Porto, Portugal; 4 Universität Hohenheim, Institut für Kulturpflanzenwissenschaften, Fg. 340a Allgemeiner Pflanzenbau, Fruwirthstr. 23, 70599 Stuttgart, Germany; Sabine.Gruber@uni-hohenheim.de

**Keywords:** food safety, legumes, microbial contamination, protein, mineral

## Abstract

Biological and vegetarian raw food products, in particular based on legume sprouts, are an increasing food trend, due to their improved nutritional value when compared to seeds. Herein, protein and mineral profiles were studied in 12 lentil varieties, with varieties Du Puy, Kleine Schwarze, Rosana, Flora, Große Rote and Kleine Späths II demonstrating the highest protein percentages. After sprouting, protein percentages increased significantly in 10 of the 12 varieties, with the highest increases ranging between 20–23% in Dunkelgrün Marmorierte, Du Puy, Große Rote and Kleine Späths II varieties. While Fe concentration was significantly decreased in three varieties (Samos, Große Rote and Kleine Späths II), Zn and Mn were positively impacted by sprouting (*p* ≤ 0.05). Magnesium concentration was not affected by sprouting, while Ca and K had percentage increases between 41% and 58%, and 28% and 30%, respectively, in the best performing varieties (Kleine Schwarze, Dunkelgrün Marmorierte, Samos and Rosana). Regardless of the associated nutritional benefits, issues pertaining to sprouts microbiological safety must be ensured. The best results for the disinfection protocols were obtained when combining the seed treatment with SDS reagent followed by an Amukine application on the sprouts, which did not affect germination rates or sprout length. The increasing levels of sprout consumption throughout the world require efficient implementation of safety measures, as well as a knowledge-based selection for the nutritional quality of the seeds.

## 1. Introduction

The consumption of legume seeds and germinated sprouts is increasing, being considered “functional foods” due to their increased nutrient availability and bioactive compounds [1,2]. Furthermore, sprouting the seeds has beneficial effects over seed quality, namely increasing digestibility and reducing the content of resistant starch and anti-nutritive compounds [3].

Lentil (*Lens culinaris* L.), in addition to having high protein content, low caloric value and high levels of essential nutrients such as folate, vitamin C and fibre [4], has as an advantage, when compared to other legumes and cereals, of very low phytic acid concentration [5], as well as high total phenolic levels [6]. Hence, this legume is a good source of amino acids, nutrients and high-quality protein [7]. Additionally, studies link lentil consumption with decreased body weight and body fat [8] and antihypertensive function [9].

Although legumes are considered a healthy option, over the years there have been pathogenic outbreaks associated with the consumption of seeds and raw sprouts [10,11,12]. Many of these outbreaks occur following seed and seed sprout consumption contaminated with *Escherichia coli*, *Salmonella* spp. and sometimes *Listeria monocytogenes* [11,13]. For example, in 2011 an *E. coli* outbreak in Germany was originated from contaminated seeds from Egypt. Another outbreak that occurred in Canada in 2005, linked to the consumption of contaminated sprouts, resulted in 600 people infected [14]. These cases demonstrate that seeds and sprouts can be easily contaminated, as also shown in a recent study where *L. innocua*, *Salmonella* spp. and coliform bacteria were found in microgreens and seed sprouts in Latvia [15]. Moreover, consumer demand for food that marketed as natural has resulted in the reduction of pesticides and other compounds, making it more likely to encounter infected seeds.

It is therefore necessary to ensure the safety of these products by developing methods that the consumer can use in the washing and disinfection of these foods at home, because they are usually consumed raw or lightly cooked [15,16]. Common household disinfection methods include hot water, acetic acid and Amukine applications, whereas sodium dodecyl sulphate (SDS), sodium hypochlorite and ethanol are common reagents used in laboratory applications for material disinfection [17].

The main objectives of this study were to comprehend the impact of sprouting on the nutritional profile of a collection of 12 lentil varieties and to understand the efficacy of different disinfection methods at eliminating *E. coli* and *Salmonella* spp. in both seeds and seed sprouts, without compromising germination percentage and sprout length.

## 2. Materials and Methods

### 2.1. Plant material and Seed Germination

A field trial was set up at the research station Kleinhohenheim that is located in south-west Germany near the city of Stuttgart (48°44’N, 9°11’O; 435 m a.s.l.). The research station has been managed organically since 1993. The climate is temperate (Cfb according to Köppen-Geiger classification [18]) and, during the experiment, which occurred from 6th April 2016 to 28th July 2016, the mean temperature was 14.9 °C and the sum of precipitation was 264 mm. At the summer solstice, there were 16 h 7 min between sunrise and sunset at the location. The soil type of the field was a loess-born silty loam with good drainage, with a pH of 6.3, total mineral N of 48 kg/ha and 9 mg/100 g of P_2_O_5_, 18 mg/100 g of K_2_O and 12 mg/100 g of Mg. In April 2016, the area was prepared for seeding by a rotor harrow and 12 lentil germplasms were sown by hand in double rows of 1 m length with a row spacing of 15 cm, and a target plant density of 240 lentil plants m^−2^. To avoid lodging, a fence of mesh wire was installed for all plots where the lentils fixed themselves by their tendrils. During the experimental period, the plants were healthy and did not present any signs of diseases or infestations. Twelve different lentil varieties were harvested and stored at 4 °C until further studies: 1—Dunkelgrün Marmorierte; 2—Du Puy; 3—Thessalia; 4—Dimitra; 5—Samos; 6—Kleine Schwarze; 7–Rosana; 8—Flora; 9—Santa; 10—Große Rote; 11—Kleine Rote; 12—Späths Alblinse II ‘Die Kleine’.

Seeds were germinated according to the protocol used by Shanmugam et al. [19]. Briefly, 50 seeds were placed in a beaker, covered with 70 % ethanol, and left for five minutes with agitation. The ethanol was discarded and a solution of 1.2% sodium hypochlorite and 0.02% SDS was added to cover the seeds and was left for 15 minutes with agitation. The solution was discarded and seeds were rinsed five times with deionized water. Afterwards, the seeds were germinated in Petri dishes with two bottom layers of paper filter moistened with deionized water, in the dark, at room temperature. At the end of five days, the lentil sprouts were stored in liquid nitrogen and then lyophilized for the nutritional analyses. This experiment was conducted in triplicate for all 12 varieties. To calculate the percentage of germination the following formula was applied:

(1) $$\%\;germination=\frac{sprouts}{total\;seeds}*100$$

### 2.2. Nutritional Analysis

Samples of seeds and seed sprouts of the 12 lentil varieties (*n* = 3) were analysed for minerals and protein. Mineral analysis determination was performed as described by Santos et al. [20]. The minerals analysed were iron (Fe), zinc (Zn), manganese (Mn), magnesium (Mg), calcium (Ca) and potassium (K). Briefly, 200 mg of the dried seed or seed sprout material was mixed with 6 mL of 65% HNO_3_ and 1 mL of 30% H_2_O_2_ in a Teflon reaction vessel and heated in a SpeedwaveTM MWS-3+ (Berghof, Germany) microwave system. Digestion procedure was conducted in five steps, consisting of different temperature and time sets: 130 °C/10 min, 160 °C/15 min, 170 °C/12 min, 100 °C/7 min, and 100 °C/3 min. The resulting clear solutions of the digestion procedure were then brought to 50 mL with ultrapure water for further analysis. Mineral concentration determination was performed using the ICP-OES Optima 7000 DV (PerkinElmer, Waltham, MA, USA) with radial configuration.

Seeds and seed sprouts were analysed for crude protein concentration (N × 5.28) using a Leco nitrogen analyzer (Model FP-528, Leco Corporation, St. Joseph, MO, USA).

### 2.3. Preparation of Inocula and Seed Inoculation

To ensure seed contamination for optimizing seed disinfection methods, seeds of the *Rosana* variety were inoculated according to the protocol used in [4]. In short, two solutions of 200 mL of Buffered Peptone Water nutrient medium (BPW) were prepared with 2 mL of *E. coli* and 2 mL of *Salmonella* spp. inocula. Using these solutions, 60 g were inoculated with *E. coli* 1.0 × 10^−8^ UFC/ml and another 60 g were inoculated with *Salmonella* spp. 1.0 × 10^−8^ UFC/ml. Seed samples were incubated for five minutes with gentle agitation. An additional 60 g of seeds was incubated with no bacteria inocula as control. After decanting the supernatant, the seeds were placed on a tray lined with filter paper and dried in a biosafety cabinet at room temperature (approximately 20 °C) for eight hours to determine the seed bacterial load.

### 2.4. Seed Contamination Evaluation

Seeds—inoculated and control—were placed in different sterile stomacher bags with buffered peptone water (BPW) until making a 1:10 dilution. Afterwards, the seed samples went to the stomacher in cycles of approximately 10 seconds at a time until a total of approximately one minute. The bacterial load on untreated and treated *Rosana* seeds was determined by the plate count method. McConkey Agar was used to plate *E. coli* and Rapid Agar *Salmonella* was used to plate *Salmonella* spp. Plates were incubated at 37 °C for 24 h. The results obtained were then converted to UFC/mL by using the following formula:

(2) $$UFC/mL=\frac{Number\;of\;colonies*dilution\;factor}{volume\;of\;culture\;plate}$$

### 2.5. Seed and Seed Sprout Disinfection Methods

Two methods of seed disinfection were compared: (1) 70% ethanol for five minutes followed by 15 minutes of a solution of 1.2% sodium hypochlorite and 0.02% SDS; and (2) hot water treatment [4], which consisted of placing the seeds in deionized water at 80 °C for 90 s, followed by drying the seeds on a sterile paper filter.

For the first method, 20 g of inoculated seeds with *E. coli* and *Salmonella* spp. were disinfected and then the bacterial load on the seed was determined by the plate count method. The same procedure was followed when testing the hot water method. Afterwards, the seeds were germinated following germination protocol described previously. The resulting sprouts were measured and went through two more disinfection protocols: cleansing with water; and treatment with Amukine, following manufacturer instructions: 15 min, 50 mL for 2.5 L of water. After these two procedures, the microbial charge on the seeds and seed sprouts was determined by the plate count method.

### 2.6. Statistical Analysis

All data were analysed with GraphPad Prism version 6.00 for Mac OS X (GraphPad Software, La Jolla, CA, USA [21]) using Tukey’s test.

## 3. Results and Discussion

### 3.1. Germination Efficiency

Germination efficiency and nutritional analyses were performed in the 12 lentil varieties to select the best performing seed variety for the microbiology study.

**Figure 1:**
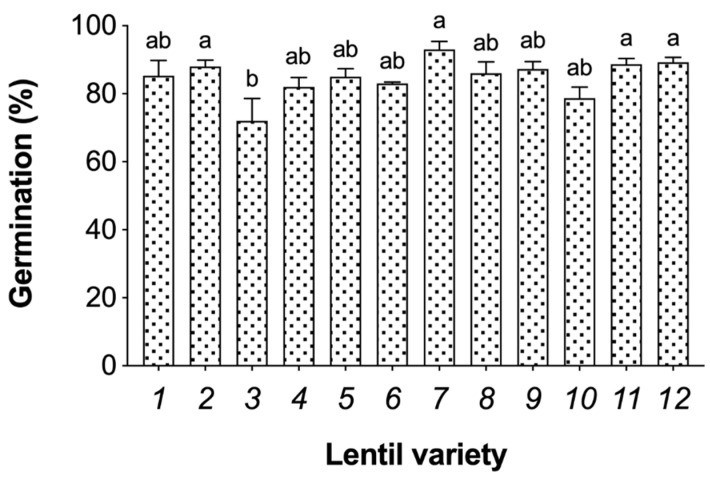
Germination rates of the 12 lentil varieties: 1—Dunkelgrün Marmorierte; 2—Du Puy; 3—Thessalia; 4—Dimitra; 5—Samos; 6—Kleine Schwarze; 7—Rosana; 8—Flora; 9—Santa; 10—Große Rote; 11—Kleine Rote; 12—Kleine Späths II. The bars represent means ± SE (*n* = 3); different letters indicate significant differences (*p* ≤ 0.05) by Tukey’s Test.

Germination is a bioprocess in which dry pulse seeds move from a dormant state to a metabolically and cellularly active state [22]. In Figure 1, the germination rates of each lentil variety were assessed and Du Puy (88%), Rosana (93%), Kleine Rote (88%) and Kleine Späths II (89%) were the highest performing varieties.

### 3.2. Nutritional Analysis

In terms of physicochemical properties, unlike other pulses, such as chickpea, fava bean or pea, lentil germplasms are relatively stable and their pasting properties or hydration capacity vary little across different accessions/varieties [23,24]. This is relevant since these characteristics are significantly correlated to the nutritional quality of the seeds, herein analysed.

In the case of protein concentration, in the present study, a significant variation was found between the lentil varieties (*p* ≤ 0.05), and this intraspecific variation was also observed in a different group of 12 lentil varieties analysed in a different study [25]. The mean protein concentration amongst seeds was 24.4% and the highest measured varieties (with values above the mean) were Du Puy, Kleine Schwarze, Rosana, Flora, Große Rote, Kleine Späths II.

**Figure 2:**
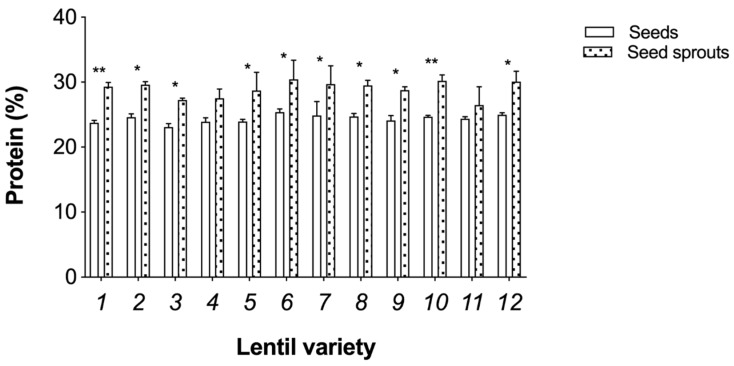
Protein concentration (%) of the seeds and seed sprouts of 12 lentil varieties: 1—Dunkelgrün Marmorierte; 2—Du Puy; 3—Thessalia; 4—Dimitra; 5—Samos; 6—Kleine Schwarze; 7—Rosana; 8—Flora; 9—Santa; 10—Große Rote; 11—Kleine Rote; 12—Kleine Späths II. Bars represent means ± SE (*n* = 3). * and ** indicate significant differences between seeds and seed sprouts at *p* ≤ 0.05 and *p* ≤ 0.01 respectively, by ANOVA using Tukey’s Test.

Furthermore, after seed germination, the protein concentration was highly increased in all lentil varieties (Figure 2), and mean protein concentration in seed sprouts was 29%. For varieties with significant variation between protein levels in seeds and seed sprouts, the ones with the highest percentage increases were Dunkelgrün Marmorierte (23%), Du Puy (20%), Große Rote (22%) and Kleine Späths II (20%). Comparative studies using different pulses also showed that lentil has the highest protein content and that total protein values increase after germination [22,25]. In the present study, protein was measured as total N content, which has been reported to remain unaltered by germination [26]. Thus, the higher values of protein here reported are promising but should be confirmed in future studies.

Regarding seed and seed sprout mineral concentrations, six nutrients were selected for the present analysis. The selected micronutrients were Fe, Zn and Mn and the macronutrients Mg, Ca and K (Table 1).

As observed in other studies [27], Fe concentration values were on average ~50 µg/g (Table 1). The lentil varieties with highest Fe concentration (both in seeds and seed sprouts) were Thessalia, Dimitra, Rosana, Flora and Kleine Rote. Germination process leads to a significant decrease in Fe concentration of Samos (36%), Große Rote (11%) and Kleine Späths II (14%) varieties. This effect of germination in Fe concentration has been reported in previous studies using different legumes seeds, namely, soybean and kidney bean [28,29]. These studies showed that the seed Fe concentration decrease was counterbalanced by a major improvement in the availability of Fe. The concentration of the micronutrients Zn and Mn, on the contrary, were shown to be positively impacted by germination, as obtained here (Table 1).

More specifically, of the 12 varieties under analysis, only five did not register a significant increase in Zn concentration after sprouting. In general, the varieties with higher Zn concentration were Thessalia, Dimitra, Kleine Schwarze and Kleine Rote. Higher Zn concentration increases (*p* ≤ 0.0001) were found in Dunkelgrün marmorierte (29%), Du Puy (21%), Samos (22%) and Kleine Späths II (24%) varieties. In regards to Mn concentrations (Table 1), Thessalia, Große Rote and Kleine Rote demonstrated the highest both in seeds and seed sprouts; and the varieties that presented a significant increase after germination were Du Puy (108%), Rosana (64%), Große Rote (52%) and Kleine Rote (42%). Legume germination is associated with a drastic reduction in phytate content, which in seeds bind with minerals, forming insoluble complexes, making them unavailable [29,30].

Amongst the analysed macronutrients, Mg concentration was not affected by germination (Table 1), similarly to what was found in a study using soybean seeds [29]. Calcium concentration, on the other hand, was reported to increase approximately 55% in legume seeds after germination [30,31], as well as its bioavailability [28]. In the present study, the varieties with the highest Ca concentration (Samos and Kleine Rote) did not show variations after germination. The varieties in which Ca concentration was significantly increased were Dunkelgrün Marmorierte (58%), Du Puy (56%), Kleine Schwarze (41%) and Rosana (49%).

In terms of K concentrations, it was reported that soybean seed sprouts present a concentration five-times higher when compared to dry seeds [29]. In the present study (Table 1), the varieties with the highest K concentration were Thessalia, Kleine Schwarze, Santa and Große Rote. Seed K concentration has been identified as a possible marker for germination capacity due to its role in initiating the imbibition of water and facilitating the associated physiological processes [32,33]. Here, the varieties with the highest K concentration (Table 1) are not amongst the ones with significantly higher germination rates (Figure 1); however, significant differences amongst varieties were few. Only Rosana variety presented significant increases (30%) in K concentration after germination (Table 1).

As all 12 varieties were grown in the same field conditions, the differences detected in this study are mainly genotypic. Interestingly, Dunkelgrün Marmorierte and Du Puy varieties, that share the same genetic background, showed no significant differences for the analysed factors, both in the seeds and seed sprouts. Knowledge on the correlation between genotypic variation and nutritional traits can contribute to future breeding programs as well as for a targeted selection of the most appropriate varieties for human consumption.

Between the four varieties with the highest germination rate—Rosana, Kleine Späths II, Kleine Rote and Du Puy—and based on their percentage of protein and potential for mineral increase after germination, the following studies proceeded with the Rosana variety.

### 3.3. Microbial Counting and Disinfecting Methods

Firstly, the impact of the seed disinfection treatments on lentil germination percentage was tested (Table 2). It was found that in general, the germination efficiencies for all treatments and controls were high, with most seeds germinating at over 90%. In general, there was no negative impact of disinfection on germination efficiency, albeit the hot water treatment for *Salmonella* inoculated seeds seems to have lowered the germination values by about 32.6% when compared to the non-disinfected non-inoculated seeds and non-disinfected and inoculated seeds.

In order to test the different disinfection treatments, the seeds were artificially inoculated with *Salmonella* spp. and *E. coli.* As shown in Table 3, disinfection with only hot water was efficient for reducing *E. coli* (from 2.7 × 10^8^ to 2.7 × 10^7^ UFC/mL), as was SDS treatment for *Salmonella* spp. (from 1.1× 10^8^ UFC/mL to ≤1.0 × 10^8^ UFC/mL). Regarding sprouts, the reduction was more accentuated when the combination of Amukine and SDS was applied, reducing 2 logs (corresponding to a 99% reduction of bacterial load) for *E. coli* when compared to sprouts inoculated with no disinfection and 1 log logs (corresponding to a 90% reduction of bacterial load) for *Salmonella* spp.

In 1999, the U.S. Food and Drug Administration recommended the utilization of 20,000 ppm of calcium hypochlorite for seed disinfection [34] and this is the method commonly used by sprout manufacturers. However, this treatment was considered potentially hazardous to the environment and industrial workers, and new efficient strategies are needed to improve sprout safety [16].

The products selected for this experiment are of easy access and are usually utilized for disinfection since they have chemical constituents, such as hypochlorite (found in common household bleach and Amukine) and ammonia, that act by denaturing bacterial proteins, similar to what happens when exposed to high temperatures resulting in bacterial death [35], or when exposed to ethanol and sodium hypochlorite. Here, some combinations of these treatments were tested to mimic and improve the disinfections done in a domestic environment.

As shown in Table 1, in general, the germination rate was not affected by the treatments or the inoculation with the pathogens, as they presented similar germination rates. However, seeds inoculated with *Salmonella* spp. treated with only hot water showed lower rates of germination, of about 65%. This could denote higher sensitivity of the seeds to this method. Also, the length of the sprouts was not affected overall (data not shown). Hence, these treatments could be applied on the seeds with relatively little impact on germination and growth processes.

The effect of the disinfection treatments on the bacterial load was also evaluated. In this study, the reduction of microorganisms was expected, not only on the seeds but also after germination. Overall, the treatment more suitable for microorganism reduction was disinfection of seeds with SDS followed by Amukine treatment after germination.

In the case of seed decontamination, the protocol that showed better results—less microbiological growth—in *E. coli* contamination was the one used in [4]. Treatment with hot water reduced the growth of *E. coli*, showing that heat inactivation is effective for this pathogen, as also observed elsewhere [36]. On the other hand, *Salmonella* was more susceptible to SDS treatments, which reduced colony count to below the detection levels.

Concerning seed sprouts, the same was verified. For *E. coli* hot water was more effective than SDS as in Barampuram et al. [37] and for *Salmonella* spp. the best results were obtained with a combination of ethanol, sodium hypochlorite and SDS. This could be due to the fact that *Salmonella* spp takes longer to be inactivated by the heat, making treatment with SDS more suitable for this pathogen.

Considering the seeds that suffered treatment before and after germination, Amukine combined with SDS presented a reduction of 1 log for *Salmonella* spp. and 2 logs for *E. coli,* being the better treatment for disinfection. Amukine works by denaturation of the cell’s proteins making them inactive while washing the sprouts with water only dilutes the microorganism existent in the plant, making this treatment not enough for proper disinfection.

## 4. Conclusions

Lentil is an affordable source of dietary plant-based protein and other nutrients and consuming it as a sprout has several associated health advantages, as this process induces improved chemical alterations. In the present study, 12 lentil varieties were analysed for their germination capacity and nutritional composition. Germination rates ranged between 72% and 93% and Du Puy, Rosana, Kleine Rote and Kleine Späths II were the highest performing varieties. In terms of protein, Du Puy, Kleine Schwarze, Rosana, Flora, Große Rote and Kleine Späths II had the highest percentages. Of these, Du Puy, Große Rote and Kleine Späths II showed a significant increase in protein concentration after germination. Furthermore, the germination process also impacted micronutrient levels, especially Zn and Mn, that were commonly increased in Du Puy variety. In regards to macronutrients, Ca was increased in Dunkelgrün Marmorierte, Du Puy, Kleine Schwarze and Rosana.

To assess microbiological safety of the sprouts, different disinfection treatments were tested, both for seeds and seed sprouts of Rosana variety. We also concluded that the most effective method for disinfection was the application of 70% ethanol, 1.2% sodium hypochlorite and 0.02% SDS before germination and Amukine afterwards. Seed decontamination for sprout consumption remains a challenge to the sprout industry. Additionally, under common household conditions, seed disinfection is usually neglected, increasing the risk of illness associated with foodborne pathogens in contaminated sprouts.

## Figures and Tables

**Table 1:** Iron (Fe), zinc (Zn), manganese (Mn), magnesium (Mg), calcium (Ca) and potassium (K) concentration (µg/g) of the seeds and seed sprouts of 12 lentil varieties and correspondent *P* values of the differences between them by ANOVA using Tukey’s Test.

Lentil Varieties		Nutrient Concentration (µg/g)											
		Fe	*p* Value	Zn	*p* Value	Mn	*p* Value	Mg	*p* Value	Ca	*p* Value	K	*p* Value
Dunkelgrün Marmorierte	Seeds	38 ± 0.3	n.s.	31 ± 0.07	<0.0001	11 ± 0.4	n.s.	10005 ± 23	n.s.	681 ± 23	0.0013	9994 ± 308	n.s.
	Sprouts	37 ± 0.1		40 ± 0.9		13 ± 1.8		952 ± 11		1079 ± 73		12,130 ± 372	
Du Puy	Seeds	37 ± 0.9	n.s.	30 ± 0.04	<0.0001	11 ± 0.07	<0.0001	991 ± 22	n.s.	619 ± 36	0.0109	9730 ± 303	n.s.
	Sprouts	39 ± 0.3		36 ± 0.2		23 ±4.1		891 ± 18		964 ± 36		10,023 ± 187	
Thessalia	Seeds	50 ± 0.2	n.s.	49 ± 0.1	n.s.	17 ± 0.2	n.s.	1148 ± 48	n.s.	933 ± 40	n.s.	10,769 ± 889	n.s.
	Sprouts	53 ± 0.5		48 ± 0.4		18 ± 0.5		1247 ± 104		774 ± 66		10,830 ± 394	
Dimitra	Seeds	59 ± 0.3	n.s.	42 ± 0.2	n.s.	13 ± 0.3	n.s.	1155 ± 101	n.s.	1040 ± 38	n.s.	10,296 ± 641	n.s.
	Sprouts	63 ± 0.8		44 ± 0.6		14 ± 0.8		928 ± 25		866 ± 35		10,830 ± 705	
Samos	Seeds	53 ± 0.2	<0.0001	39 ± 0.3	<0.0001	13 ± 0.2	n.s.	1067 ± 36	n.s.	999 ± 64	n.s.	9623 ± 303	n.s.
	Sprouts	34 ± 1.6		48 ± 0.8		14 ± 0.6		1039 ± 45		1138 ± 52		12,299 ± 383	
Kleine Schwarze	Seeds	47 ± 0.3	n.s.	44 ± 0.6	<0.0001	14 ± 0.3	n.s.	1201 ± 83	n.s.	754 ± 70	0.0350	11,050 ± 375	n.s.
	Sprouts	50 ± 1.7		51 ± 0.3		10 ± 0.4		1043 ± 25		1067 ± 68		10,292 ± 169	
Rosana	Seeds	68 ± 1.5	n.s.	35 ± 0.5	n.s.	12 ± 0.5	0.0082	947 ± 70	n.s.	775 ± 62	0.0026	10,149 ± 68	0.0419
	Sprouts	64 ± 3.1		38 ± 0.6		20 ± 0.8		1017 ± 52		1156 ± 113		13,154 ± 1043	
Flora	Seeds	51 ± 0.3	n.s.	37 ± 0.4	n.s.	12 ± 0.7	n.s.	1107 ± 73	n.s.	851 ± 62	n.s.	9414 ± 807	n.s.
	Sprouts	52 ± 0.4		39 ± 0.09		12 ± 0.5		1010 ± 55		759 ± 55		11,350 ± 491	
Santa	Seeds	43 ± 0.4	n.s.	36 ± 1.5	n.s.	13 ± 0.9	n.s.	1168 ± 67	n.s.	766 ± 4	n.s.	10,905 ± 668	n.s.
	Sprouts	43 ± 0.3		36 ± 0.2		16 ± 0.6		1044 ± 72		668 ± 30		10,128 ± 192	
Große Rote	Seeds	51 ± 0.2	0.0374	38 ± 1.4	0.0186	15 ± 2	0.0079	1006 ± 47	n.s.	864 ± 52	n.s.	10,905 ± 668	n.s.
	Sprouts	45 ± 0.5		42 ± 1.1		23 ± 0.6		1061 ± 27		980 ± 35		10,128 ± 192	
Kleine Rote	Seeds	66 ± 0.2	n.s.	43 ± 0.3	0.0195	17 ± 0.6	0.0167	1084 ± 17	n.s.	1062±70	n.s.	10,423 ± 849	n.s.
	Sprouts	63 ± 2.2		47 ± 1.1		25 ± 1.1		1048 ± 28		1146 ± 62		10,216 ± 308	
Kleine Späths II	Seeds	44 ± 0.2	0.0152	38 ± 0.2	<0.0001	11 ± 1.2	n.s.	1224 ± 121	n.s.	623 ± 52	n.s.	10,121 ± 211	n.s.
	Sprouts	38 ± 0.9		47 ± 0.1		16 ± 1.5		1075 ± 54		803 ± 21		10,473 ± 425	

n.s. not significant.

**Table 2:** Germination percentage after disinfection treatments in lentil seeds (variety Rosana) inoculated with *E. coli*, *Salmonella* spp. and control.

Treatments	% Germination		
	*E. coli*	*Salmonella* spp.	Control
No disinfection/without inoculation	-	-	98.8
No disinfection/after inoculation	99.3	96.9	-
SDS disinfection	96.3	96.0	97.3
Water (80 °C) disinfection	95.5	65.3	82.3

**Table 3:** Microbial counting after disinfection treatments (UFC/mL) of lentil seeds and sprouts.

Treatments	Seeds		Sprouts	
	*E. coli*	*Salmonella*	*E. coli*	*Salmonella*
**No disinfection/without inoculation**	≤1.0 × 10^8^	≤1.0 × 10^8^		
**No disinfection/with inoculation**	2.7 × 10^8^	1.1 × 10^8^	1.8 × 10^8^	1.4 × 10^8^
**SDS disinfection**	1.1 × 10^8^	≤1.0× 10^8^	11.4 × 10^8^	2.7 × 10^8^
**Water (80 °C) disinfection**	2.7 × 10^7^	>3.0 × 10^8^	2.1 × 10^8^	1.2 × 10^8^
**Water (80 °C) + H_2_O rinse**			9.0 × 10^7^	9.4 × 10^7^
**Water (80 °C) + Amukine**			9.8 × 10^7^	8.0 × 10^7^
**SDS + H_2_O rinse**			6.0 × 10^7^	9.8 × 10^8^
**SDS + Amukine**			3.9 × 10^6^	5.6 × 10^7^
